# Thermal, Microstructural, and Mechanical Analysis of Complex Lattice Structures Produced by Direct Energy Deposition

**DOI:** 10.3390/ma17122813

**Published:** 2024-06-09

**Authors:** David G. Andrade, Carlos Zhu, Hélio C. Miranda, Dulce M. Rodrigues

**Affiliations:** 1 ISISE, ARISE, Department of Civil Engineering, University of Coimbra, 3030-788 Coimbra, Portugal; david.andrade@uc.pt (D.G.A.); carlos.zhu@student.dem.uc.pt (C.Z.); 2 CEMMPRE, ARISE, Department of Mechanical Engineering, University of Coimbra, 3030-788 Coimbra, Portugal; 3 Department of Metallurgical and Materials Engineering, Federal University of Ceara, Fortaleza 60430-140, Brazil; hmiranda@ufc.br; 4 ISISE, ARISE, Department of Mechanical Engineering, University of Coimbra, 3030-788 Coimbra, Portugal

**Keywords:** lattice structures, auxetic, additive manufacturing, WAAM, thermal cycles, microstructure, hardness

## Abstract

Lattice structures have gained attention in engineering due to their lightweight properties. However, the complex geometry of lattice structures and the high melting temperature of metals present significant manufacturing challenges for the large-scale fabrication of these structures. Direct Energy Deposition (DED) methods, such as the Wire Arc Additive Manufacturing (WAAM) technique, appear to be an interesting solution for overcoming these limitations. This study provides a detailed analysis of the manufacturing process of carbon steel lattice structures with auxetic geometry. The study includes thermal analysis using infrared thermography, microstructural characterization through metallography, and mechanical evaluation via hardness and mechanical testing. The findings reveal the significant impact of heat input, thermal cycles, and deposition sequence on the morphology and mechanical properties of the lattice structures. Fast thermal cycles are related to areas with higher hardness values, smaller strut diameters, and porous formations, which shows that controlling heat input and heat dissipation is crucial for optimizing the properties of lattice structures produced using WAAM.

## 1. Introduction

Lattice structures, which consist of slender elements combined in specially designed patterns, have recently gained a lot of interest from the scientific community, due to the possibility of using them to create unique components, which are inherently lightweight and may display a tailored mechanical performance, such as a negative Poisson’s ratio [1,2]. The potential applications of the lattice structures span a wide range of industries, including biomedical [3], aerospace [4], military [5], and construction [6,7]. However, for the industrial production of such structures to become feasible, the ability to produce large components becomes imperative. Wire Arc Additive Manufacturing (WAAM) [8,9] has emerged as an optimal process for solving this problem thanks to its capability to rapidly produce large structures, compared to other metal additive manufacturing (MAM) techniques, such as Selective Laser Melting (SLM) [10] or Laser Powder Bed Fusion (LPBF) [11].

Examples of the use of WAAM in the production of large lattice structures are, for example, the works conducted by Radel et al. (2019) [12] and Zheng et al. 2022 [4]. Radel et al. (2019) [12] utilised WAAM to produce a lattice structure for a cantilever beam, while Zheng et al. 2022 [4] fabricated a thermal insulation and load-bearing component for hypersonic vehicles. These studies show that the methodologies used for fabricating lattice structures employing WAAM diverge from the typical layer-by-layer approach used for fabricating bulk solids. Alternative methodologies are used, which consist of sequentially producing the various struts of the lattice, using dot-by-dot metal deposition. The majority of the studies available in the literature on the construction of lattice structures using WAAM are primarily focused on this metal deposition technique and on the software upgrades required to optimise the production of the slender geometries. The software optimisation envisaged enhancing the morphology of the struts composing the lattices [13,14,15,16], improving the material deposition and the connections between struts [17,18,19], mitigating collisions between the part under construction and the torch [12], or implementing vision control systems [12,20].

To ensure the proper production of complex lattice structures, such as metallic auxetics, for example, it is essential to achieve a proper balance between the heat input and the material deposition. If the heat input is insufficient, it can cause important variations of the strut’s diameter along its height or the incomplete melting of the feedstock wire. In contrast, excessive heat input can lead to the collapse of the molten pool [21]. For the fabrication of lattice structures in mild steel, Abe et al. 2019 [15] recommend using heat inputs higher than 960 J and lower than 2700 J. The heat input was also found to govern the strut diameter by Xu et al. 2020 [22]. The author also stated that the metal transfer and the strut morphology are improved when the strut angle is between 45° and 90°.

The interlayer temperature is another critical parameter that significantly influences the lattice structure morphology. Wu et al. 2020 [23] and Yu et al. 2022 [21] investigated the impact of the interlayer temperature on the structs morphology in the construction of aluminium alloy lattice structures. Most of the studies on the fabrication of lattice structures using WAAM have been focused on the analysis of the morphology of the struts, neglecting the analysis of their mechanical and microstructural properties, as well as its correlation with the heat input or thermal cycles. The knowledge on the relationship between heat input and material properties, will not only help in enhancing the process efficiency, but will also help in the optimization of the lattice structures properties. As lattice structures are typically produced with lower heat inputs than those used for producing bulk components through WAAM, previous results on this subject may not directly apply to the lattice structures. Therefore, this article presents a study on the influence of the WAAM thermal fields on the microstructural, mechanical, and morphological properties of carbon steel lattice structures with auxetic geometry. The study includes the thermal analysis of the material deposition process using infrared thermography, as well as the microstructural characterisation of the deposited material. A deep analysis of the heterogeneity in mechanical properties using hardness measurements was also performed, and the results were related to the thermal and microstructural analysis. The auxeticity of the produced cells will be also tested, by performing tensile tests.

## 2. Experimental Procedure

**Figure 1:**
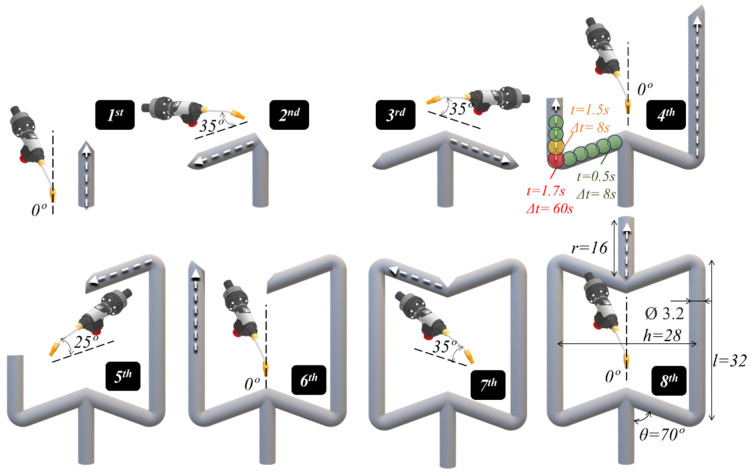
Schematic representation of the deposition strategy sequences.

In this study, lattice structures with auxetic geometry were fabricated via WAAM, using an ABB IRB 4600 robot (ABB Ltd., Zürich, Switzerland) and a Fronius CMT welding power source (Fronius International GmbH, Austria). As shown in Figure 1, where the production sequence for each of the lattice struts is illustrated, a re-entrant cell geometry was adopted in this investigation. This geometry was chosen due to the previous experience of the authors in analysing its mechanical performance [24], but also due to the important challenge in fabricating it using WAAM. The production of the cell using WAAM includes constructing vertical and inclined struts, with extreme overhang angles between them, which requires using various deposition strategies, such as vertical down, vertical up, and overhead. Moreover, the selected geometry includes producing intersections between vertical and inclined segments, which represented important challenges in terms of thermal dissipation and material deposition.

As illustrated in Figure 1, the building of the different cell segments involved eight distinct deposition sequences, performed using the dot-by-dot deposition mode of CMT. Each material deposition sequence is identified in the figure by the corresponding number. The vertical cell members produced during deposition sequence numbers 1, 4, 6, and 8 resulted from the dot-by-dot deposition of material in the vertical direction, i.e., the torch angle was identical to the segment building direction. On the other hand, for the inclined segments produced during deposition sequence numbers 2, 3, 5, and 7, the welding torch was placed with an inclination angle between 25° and 35° in relation to the member building direction. Deposition sequence number 4 consisted of the simultaneous production of the right and left vertical struts, by the alternated material deposition of the material dots in each strut. With this procedure, it was envisaged to reduce the cell production time.

**Figure 2:**
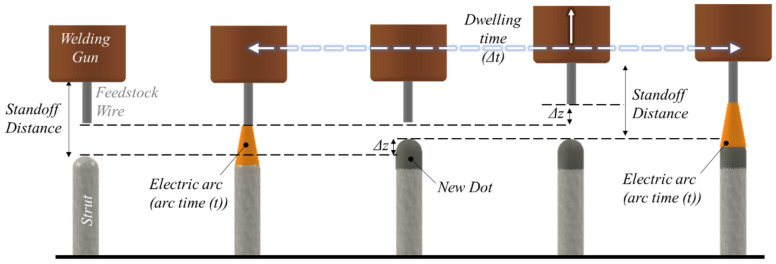
Schematic representation of the dot-by-dot material deposition strategy.

**Figure 3:**
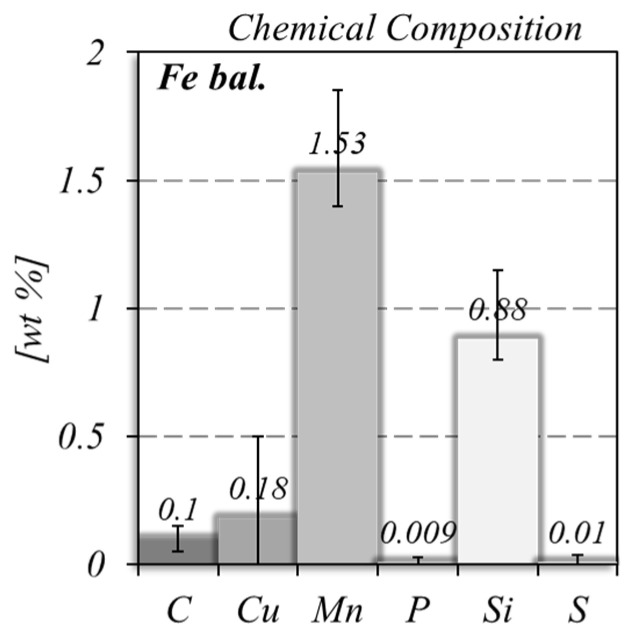
Chemical composition of the feedstock material.

As illustrated in the image corresponding to sequence number 4 in Figure 1, deposition times (*t*) of 0.5 s and dwelling times (Δ*t*) of 8 s were used for the dot-by-dot material deposition. As schematized in Figure 2, the deposition time corresponds to the arc time, during which the feedstock wire is melted and a material dot is deposited, and the dwelling time corresponds to the time between two successive material dots. During the transition between two different deposition sequences, the dwelling time was extended to 60 s, to enable the repositioning of the welding torch. For deposition sequences 4 and 8, the first two dots were set with a deposition time of 1.7 s and 1.5 s, respectively, to ensure the re-melting of the previously deposited material. Finally, the torch was moved upward (Δ*z* in Figure 2) between the deposition of two successive dots to allow for the continuous material deposition and maintenance of the standoff distance at a constant value. Displacements of 1 mm and 0.7 mm were used for the deposition of the vertical and inclined elements, respectively. The deposition parameters are summarised in Table 1 and the chemical composition of the feedstock material is shown in Figure 3, as provided by the manufacturer. These deposition parameters/procedures were set using trial and error tests to enable the production of the overhang members, using the available laboratory facilities.

The planned cell dimensions in the image corresponding to deposition sequence number 8 are represented in Figure 1, where the height (*h*), diameter (*Ø*), and length of the struts (*l* and *r*) are represented, as well as the angle (*θ*) between the vertical and inclined cell segments. Replicas of the vertical segments were also produced, along with the re-entrant auxetic cells, with the aim of analysing the thermal dissipation within each individual cell segment.

**Figure 4:**
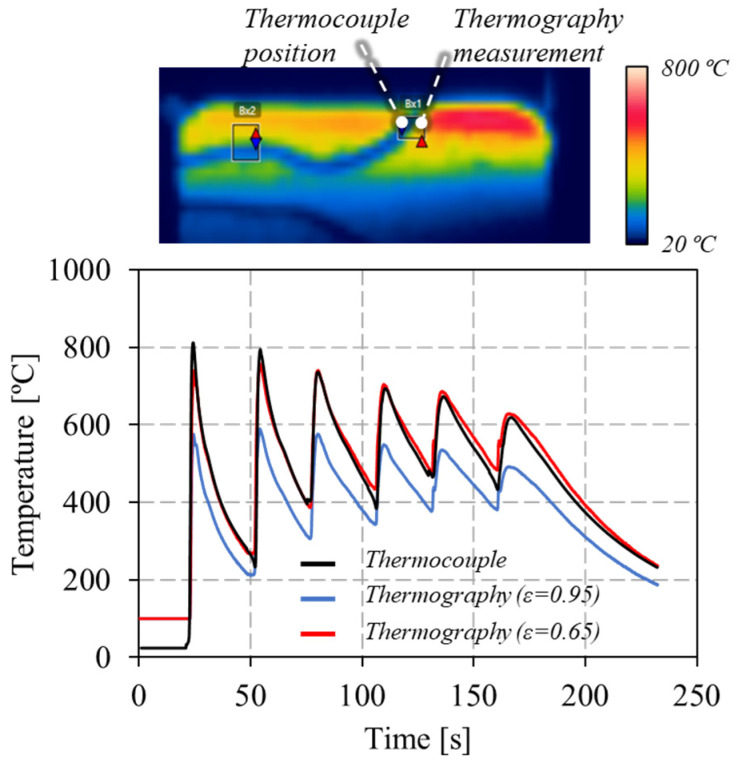
Analysis of the emissivity in the WAAM of carbon steel walls.

During the production of the auxetic cell, the thermal cycles associated with the material deposition were acquired with a FLIR A655sc thermographic camera. A constant emissivity of 0.65 was applied to determine the temperature evolution throughout the process. This value was determined during the deposition of a wall made of the same material of the lattice structure [25], by comparing the temperature readings obtained with a thermocouple welded to the wall to those recorded with the thermographic camera, as exemplified in Figure 4. In the figure, the curves representing the evolution of the temperature at the thermocouple location with time, during the deposition of several material layers, are shown. The curves correspond to the temperature registered by the thermocouple, and to the temperatures determined using the thermographic data, for two different emissivity values (0.65 and 0.95). As is shown in the graphic, the curve corresponding to the emissivity of 0.65 perfectly fits the thermocouple results.

After production, the EinScan Pro HD 3D (Shining 3D, Hangzhou, China) scanning device was utilized to capture the geometry of the cells. A optical microscope (Leica DM 4000 M LED, Wetzlar, Germany) was used to conduct a microstructural analysis. Metallographic samples were extracted from several cell locations, and were polished and etched with 2% Nital. Microhardness measurements were performed using a Microhardness Tester (Shimadzu, Kyoto, Japan), with a 200 g load and a 15 s holding time, to complement the information from the microstructural analysis. The mechanical characterisation also included performing quasi-static tensile tests of the WAAM auxetic cells, to assess their auxeticity. The tests were performed at room temperature with a loading speed of 2 mm/min using a universal machine (Shimadzu AGS-X 100 kN, Kyoto, Japan).

## 3. Results and Discussion

### 3.1. Thermal Analysis

**Figure 5:**
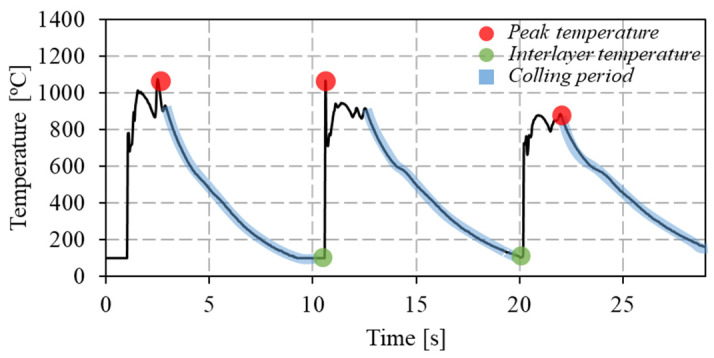
Procedures adopted for calculating the maximum and interlayer temperatures and the cooling rates.

**Figure 6:**
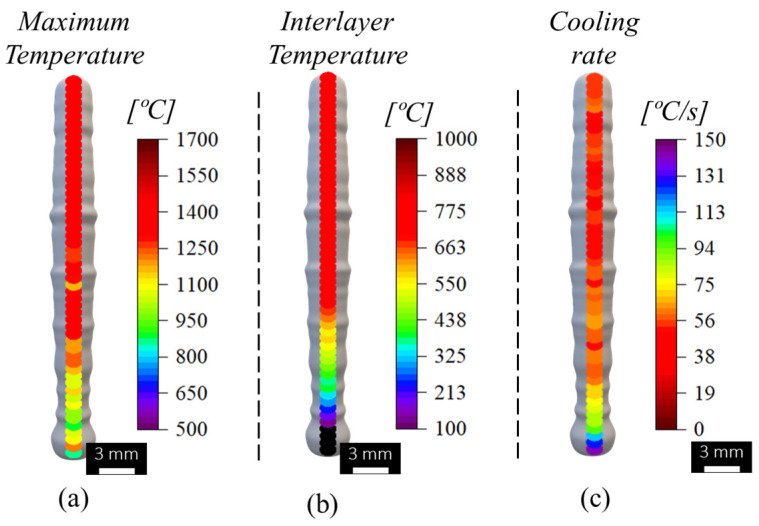
Evolution of (**a**) the maximum temperature, (**b**) interlayer temperature, and (**c**) cooling rates along the longitudinal axis of the vertical cell member.

The evolution of the maximum temperature, interlayer temperature, and cooling rates recorded during the fabrication of the individual cell segments, and determined as indicated in Figure 5, are shown in Figure 6. In Figure 5, the temperature evolution with time, during the deposition of three successive material dots, is shown. The maximum temperatures in Figure 6 correspond to the collection of the maximum temperature values registered for each dot deposition, and the interlayer temperature corresponds to the minimum temperature registered between two successive dots. The cooling rate in Figure 6 corresponds to the average of the cooling rates determined for the descending part of each temperature cycle (highlighted in blue in Figure 5).

Analysing Figure 6, it is possible to conclude that the lower maximum and interlayer temperatures were registered during the deposition of the first material dots (bottom part of the strut), due to the severe heat dissipation through the non-preheated substrate. For the middle and top parts of the segment, which were deposited over material still at an elevated temperature, the heat dissipation was slower, as shown in Figure 6c, and the maximum and interlayer temperatures were higher than that registered for the first material depositions. The figure also shows that for the middle and top regions of the strut, the dwelling time between dots was enough to ensure a constant interlayer temperature of around 800 °C. These results are similar to those registered during the deposition of bulk stainless steel walls [26].

**Figure 7:**
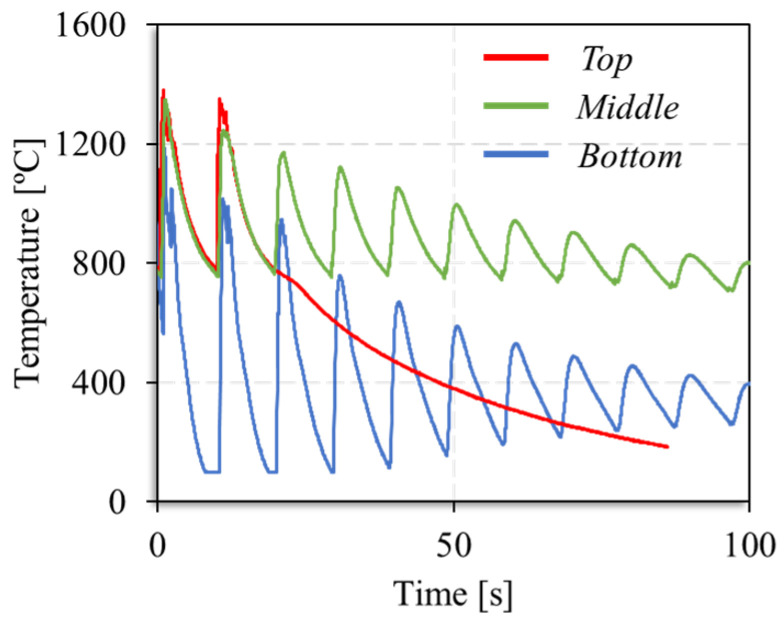
Temperature evolution during the deposition of successive material dots for the bottom, middle, and top of the vertical struts.

**Figure 8:**
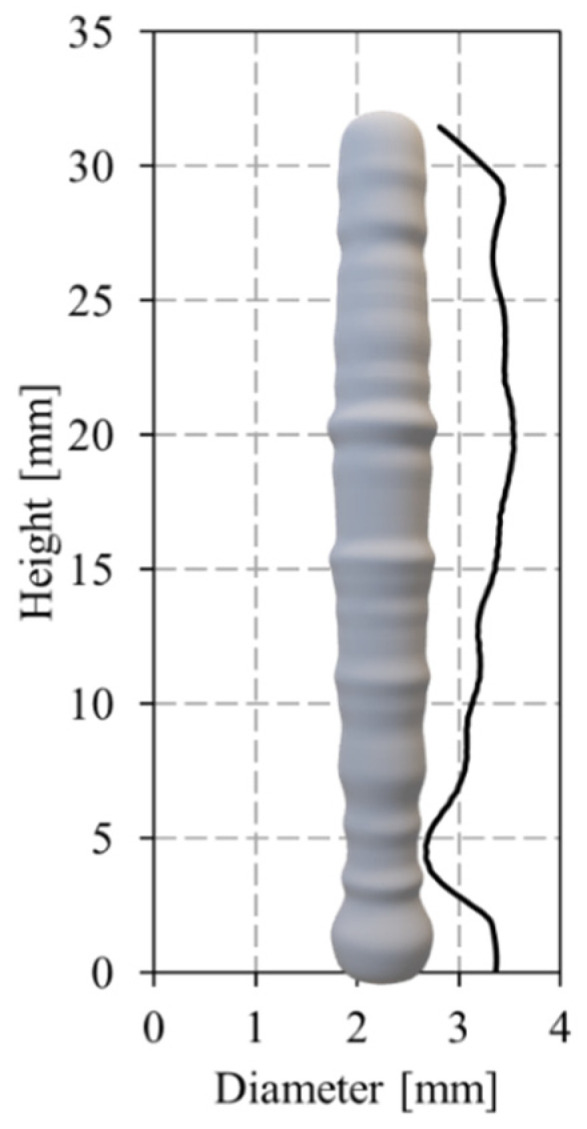
Evolution of the strut diameter with the building height.

Figure 6 also enables the conclusion that steady-state conditions in maximum and interlayer temperatures and cooling rates were reached after the deposition of eighteen material dots. This is also illustrated in Figure 7, where the temperature evolution during the deposition of successive material dots at the bottom, middle, and top of the vertical segment are shown. The time scales of the different curves were adjusted to enable the direct comparison of the temperatures registered for the different locations of the strut. The figure shows that the temperature evolution with time was similar for the dots of the top and middle parts of the segment, and very different from that registered for the dots deposited during the production of the bottom part of the strut. As a consequence of the differences in heat dissipation, the diameter of the vertical segments evolves from the bottom to the top of the segment, together with the temperature distribution, as is shown in Figure 8, where the evolution of the strut diameter with the building height is represented. The image shows that the segment diameter increases from the bottom, where the lower temperatures were registered, to the top, where the highest temperatures were registered.

**Figure 9:**
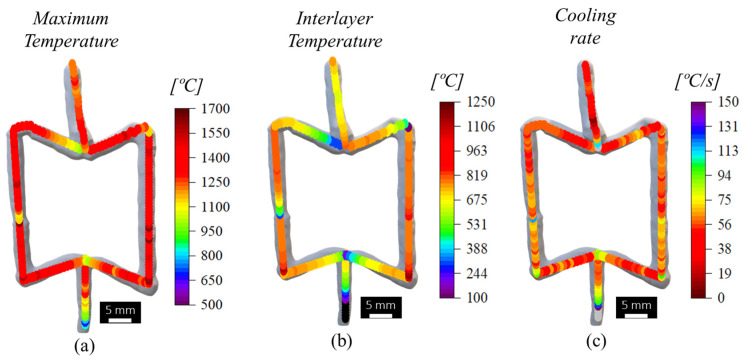
Evolution of the (**a**) maximum temperatures, (**b**) interlayer temperatures, and (**c**) cooling rates during the building of the re-entrant cell.

In Figure 9, the distribution of the maximum and interlayer temperatures registered during the fabrication of the re-entrant cell are represented, as well as the cooling rates, determined using the same procedures described for the vertical segment (Figure 5). The figure clearly shows that in a similar manner to that registered for the singular vertical segment, the cell also experienced an almost uniform temperature distribution during its fabrication. The only noticeable differences in temperatures and cooling rates were registered for the regions produced during the transition between deposition sequences 1 and 3, 4 and 5, 4 and 6, and 6 and 7, for which extended dwelling times of 60 s were used. However, for the transition between deposition sequences 2 and 4, 3 and 4, and 7 and 8, even though a similar dwell time of 60 s was used, lower temperatures were not recorded because the first two spots of sequences 4 and 8 were deposited using deposition times equal to 1.7 s and 1.5 s, respectively. These results show the important influence of the deposition time on the heat input and dissipation, and how these variables may be used to ensure steady-state thermal distribution during the construction of the cells.

### 3.2. Microstructural Characterisation

**Figure 10:**
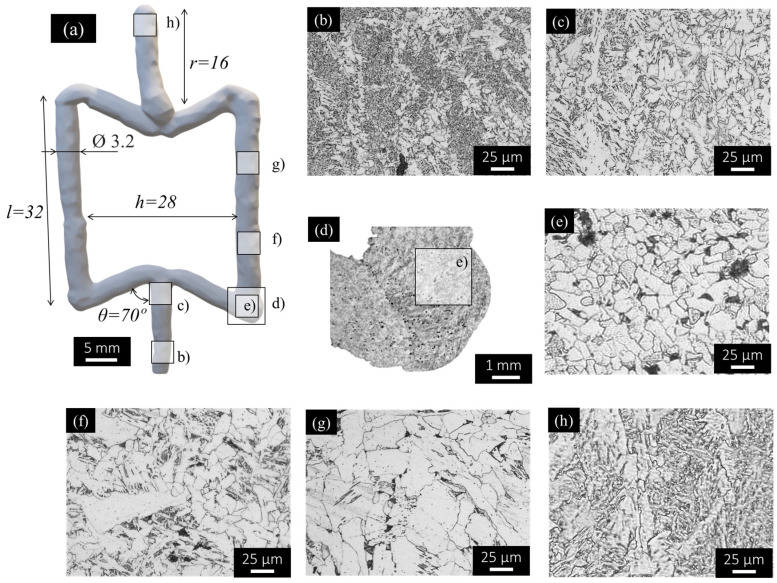
Morphology of the (**a**) re-entrant cell and (**b**–**h**) microstructural evolution at different locations.

Figure 10a shows the as-built cell geometry, acquired using laser scanning, with the main average dimensions determined for the cell displayed on it. Despite the similarities in dimensions, comparing the volume of the as-built cell with that determined for the planned geometry, shown in Figure 1, it was concluded that the as-built geometry has 24% extra mass, which results from the irregular morphology of the as-built cell. The differences between the planned and as-built geometry are mainly important in the regions corresponding to the intersection between the vertical and inclined struts, for which fabrication still needs to be improved.

In Figure 10a, rectangles identifying the locations from which the micrographs in Figure 10b–h were taken are also represented. The micrographs in Figure 10b,c represent the microstructural evolution along the first strut produced (sequence number 1), for which the steeper cooling rates were registered. The micrographs in Figure 10d,e illustrate the microstructure at the connecting zones between deposition sequences 3 and 4 and the micrographs in Figure 10f,g represent the microstructural evolution along the strut produced during deposition sequence number 4. Finally, Figure 10h represents the microstructure of the top part of the last strut being deposited.

Analysing the images, it is possible to conclude that the regions closer to the substrate (Figure 10b), which experienced severe cooling rates, are composed of refined ferrite, allotriomorphic ferrite, and bainitic grains. Moving along the build direction (Figure 10c), the same microstructures may be observed, despite coarser grain sizes. This is the result of the heat accumulation during the dot-by-dot material deposition, as was demonstrated by the thermal analysis results in Figure 6.

Analysing the images in Figure 10d,e, which correspond to the connecting region between the segments produced during sequences 3 and 4, it is possible to conclude that the extended dwelling time of 60 s conducted to severe cooling rates, since the grain sizes in these regions are smaller than in the previous ones. In this figure, it is also possible to observe small pores along the entire sample cross-section, resulting from the fast-cooling rates, which lead to the entrapment of gases when the weld pool solidifies. However, since the thermal history experienced by the deposited material along the build direction resulted in heat accumulation and steady-state heat dissipation, for the middle of the strut, coarser grains of polygonal ferrite and intergranular lamellar pearlite were, once again, registered, as shown in Figure 10f,g. Finally, as shown in Figure 10h, in the last part of the cell being deposited, a mixture of acicular ferrite, allotriomorphic ferrite, and bainite was registered, as for the bottom of the first strut. The microstructures are similar, since the upper part of the cell did not experience the thermal effect of other depositions and no recrystallization took place after the solidification of that material.

### 3.3. Mechanical Characterisation

**Figure 11:**
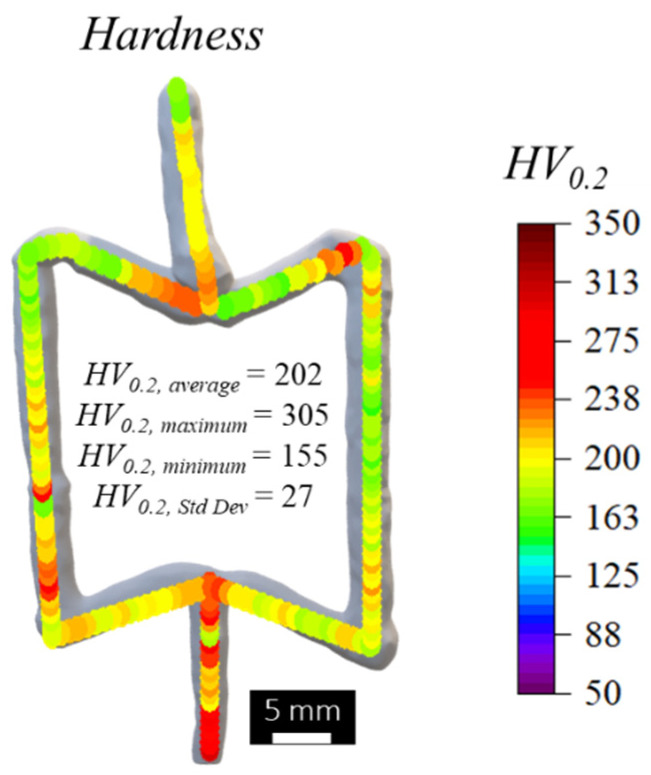
Hardness distribution map and hardness average, maximum, minimum, and standard deviation values.

The presence of a heterogeneous microstructure along the building direction of the auxetic cell may lead to a mismatch in mechanical properties between the different cell segments, potentially influencing the macroscopic behaviour of the cell and its auxeticity. The mismatch in mechanical properties among the cells was assessed by performing hardness measurements. Figure 11 shows the hardness map obtained, together with the average, maximum, minimum, and standard deviation values calculated from these hardness profiles. The results show that the hardness values had a gradient along the cell volume, decreasing from the base to the top of the cell. This is also evident for each deposition sequence, which presented higher hardness values at the beginning than at the end of the printing sequence. The average hardness of the cell was determined to be 202 HV_0.2_, with a range spanning from a maximum of 305 HV_0.2_ to a minimum of 155 HV_0.2_, resulting in a standard deviation value of 27 HV_0.2_.

The presented findings highlight the substantial influence of thermal cycles on cell microstructure and mechanical properties. The regions experiencing lower cooling rates and higher temperatures for extended periods exhibit larger grain sizes, accompanied by lower hardness values. Contrary to this, the areas near the substrate or the initially deposited regions for each deposition sequence presented higher heat dissipation, leading to refined grain structures and higher hardness values.

**Figure 12:**
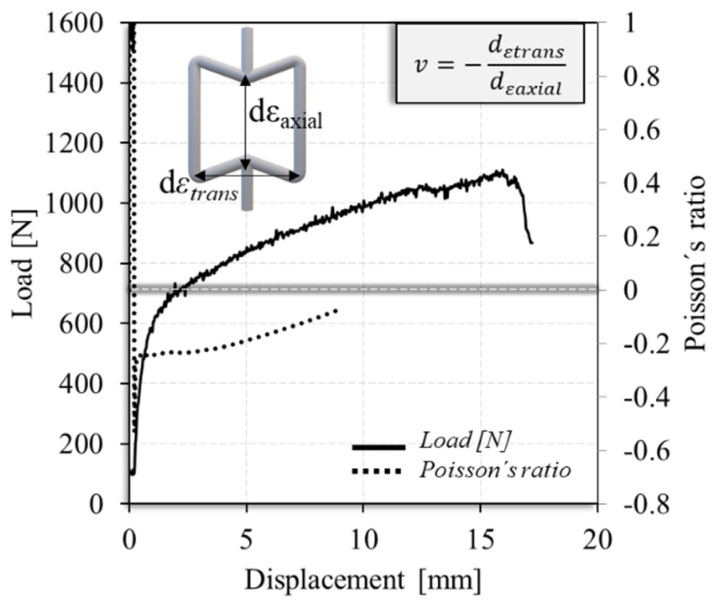
Evolution of the tensile load and Poisson’s ratio with displacement.

Figure 12 shows the load–displacement and Poisson’s ratio–displacement curves obtained for the auxetic cell, tested in tension. Poisson’s ratio was calculated by determining the ratio between the transverse and axial displacement of some selected cell nodes, as schematically represented in the figure. Although the cell presented small porous and geometrical inaccuracies, the figure shows that the cell was able to withstand large deformations of around 16 mm before failure, and displayed a negative Poisson’s ratio, which is consistent with the expected behaviour of an auxetic component. Furthermore, a more comprehensive analysis of the mechanical properties and numerical modelling of the auxetic cells fabricated using WAAM will be prepared for a future publication.

## 4. Conclusions

The present work aimed at analysing the thermal cycles and the microstructural and mechanical properties of complex lattice structures with auxetic geometry, produced using WAAM. The following conclusions were reached:

- For each strut, lower temperatures were registered during the initial material deposition due to rapid heat dissipation through the substrate or the previously deposited material. Steady-state conditions in temperature and cooling rates were achieved after the deposition of eighteen material dots. Additionally, the temperature distribution within the auxetic cell was uniform, except for the transition zones between vertical and inclined struts, emphasizing the influence of the deposition time and strategy on the thermal distribution.
- The microstructural analysis enabled the observation of structures with varying grain morphology and size along the build direction. The severe cooling rates at the cell transitions zones lead to a refined microstructure with a mixture of acicular ferrite, allotriomorphic ferrite, and bainite grains, while the regions with slower cooling resulted in coarser grains of polygonal ferrite and intergranular lamellar pearlite.
- Hardness measurements indicate a gradient in hardness values from the base to the top of the cell, with higher values at the beginning of deposition sequences. However, despite the heterogeneity in local mechanical properties and the presence of minor defects, such as porosities and geometrical irregularities, the cell displayed an auxetic behaviour when loaded in tension, withstanding significant deformation before failure.
- Based on the current findings, optimising the WAAM process by implementing a controlled deposition approach, utilising a constant interlayer temperature rather than a constant dwelling time is recommended. This adjustment aims to achieve lattice structures with a more homogeneous microstructure and mechanical properties. Furthermore, for the initial spot welds, pre-heating the previously deposited material is advised to mitigate the formation of brittle phases and defects like pores. By integrating these recommendations into the fabrication process, it is anticipated that the resulting lattice structures will exhibit an improved structural performance.

## Figures and Tables

**Table 1:** Deposition parameters and feedstock material.

**Current [A]**	120
**Voltage [V]**	13
**Wire feed rate [m/min]**	0.9
**Stick-out [mm]**	15
**Feedstock material**	ER70S-6
**Wire diameter [mm]**	1
**Shielding gas**	98% Ar + 2% CO_2_
**Flow rate [L/min]**	14

## Data Availability

The original contributions presented in the study are included in the article, further inquiries can be directed to the corresponding author.
